# Obstetrical Antiphospholipid Syndrome: From the Pathogenesis to the Clinical and Therapeutic Implications

**DOI:** 10.1155/2013/159124

**Published:** 2013-07-30

**Authors:** T. Marchetti, M. Cohen, P. de Moerloose

**Affiliations:** ^1^Laboratory of Hormonology, Maternity, Geneva University Hospitals, 1211 Geneva 14, Switzerland; ^2^Haemostasis Unit, Geneva University Hospitals, 1211 Geneva 14, Switzerland

## Abstract

Antiphospholipid syndrome (APS) is an acquired thrombophilia with clinical manifestations associated with the presence of antiphospholipid antibodies (aPL) in patient plasma. Obstetrical APS is a complex entity that may affect both mother and fetus throughout the entire pregnancy with high morbidity. Clinical complications are as various as recurrent fetal losses, stillbirth, intrauterine growth restriction (IUGR), and preeclampsia. Pathogenesis of aPL targets trophoblastic cells directly, mainly via proapoptotic, proinflammatory mechanisms, and uncontrolled immunomodulatory responses. Actual first-line treatment is limited to low-dose aspirin (LDA) and low-molecular weight heparin (LMWH) and still failed in 30% of the cases. APS pregnancies should be a major field in obstetrical research, and new therapeutics are still in progress.

## 1. Introduction

APS is an autoimmune disorder characterized by a high-risk of obstetrical complications affecting both mother and fetus [1, 2]. This condition can either be purely thrombotic, which will not be treated here, or obstetrical or it can combine both aspects of the syndrome. Clinical criteria of obstetrical APS have been revisited in Sydney in 2006 (Table 1) and include a history of three early miscarriages (<10 WG), and/or one stillbirth (>10 WG), and/or one intra-uterine growth restriction or a premature birth before 34 WG due to preeclampsia or eclampsia or placental insufficiency [3]. Furthermore, APS pregnant women have an increased risk of thrombosis [4], thrombocytopenia, and HELLP syndrome [5].

APS can be found as a single disease and is referred as “primary.” Secondary APS is associated with other autoimmune diseases, mainly systemic lupus erymathosus (SLE). Women are more commonly affected by APS than men, in primary (3,5 : 1 ratio) as well as in secondary APS (7 : 1) [6]. The prevalence of aPL is estimated to be 5% of the general population, and APS represents 0.5% [6, 7]. However, aPL is commonly found in 15% of women with recurrent pregnancy losses (RPLs), suggesting that APS is one of the most frequent acquired etiology for RPL [8].

aPL is a heterogeneous family of three autoantibodies including lupus anticoagulant (LA), anticardiolipin antibodies (aCL), and anti*β*2glycoprotein-1 antibodies (anti*β*2GP1 Abs). As *β*2GP1 seems the main antigen for aPL, anti*β*2GP1 Abs are now considered amongst the principal antibodies of the syndrome [9, 10]. 

During pregnancy, anti*β*2GP1 Abs affect trophoblastic cells directly by binding to *β*2GP1 at the surface of trophoblastic cells [11]. 

aPL have been incriminated in alteration of trophoblastic cells *via* different mechanisms. Pathogenesis of aPL in pregnancy include thrombotic mechanisms, inflammation, apoptosis and immunomodulatory molecules impairments in trophoblast [12].

Moreover, damages of other cell types such as endometrial cells by aPL during pregnancy have also been involved [13, 14].

Nowadays, pathogenic mechanisms still remain unclear. A better understanding of cellular interactions with aPL is necessary. Because first-line treatments with LDA and LMWH fail in about 30% of the cases, new specific therapeutics are in development [15]. The use of other medications is a matter of debate. Thus, hydroxychloroquine (HCQ), an old antimalarial drug used in SLE, has been shown to reduce antiphospholipid titers in the plasma of patients with persistent aPL [16] and to improve fetal outcomes in SLE-treated pregnant patients [17].

In this review of the literature, we discuss the clinical aspects of obstetrical APS on both mother and fetus sides, its pathogenesis, and current treatments as well as future treatment opportunities. In addition to another recent review on the same subject [18], we insist on new clinical and biological aspects of obstetrical APS. Infertility and infant development consequences are detailed as well as the potential impact of antibodies against domain I of *β*2GP1 on pregnancy. Moreover, special attention for catastrophic APS (CAPS) management is also given.

## 2. Obstetrical Manifestations of APS

In a European cohort of 1000 patients including 82% of APS women [19], Cervera et al. described the main clinical manifestations related to this syndrome during a 5-year follow-up (from 1999 to 2004). Obstetrical manifestations were very frequent; among them, prematurity and early pregnancy loss (as defined in Sydney's criteria) were the main clinical features (28% and 18%, resp.). 

Mean age of disease onset varies between studies (30–40 years), but women of childbearing age are mostly represented. 

In APS, pregnancy manifestations, severity of these complications, and maternofetal outcomes vary with aPL. Ruffatti et al. have shown that high titers and triple positivity for aPL were associated with both mother and fetal complications, even when treatment was well conducted [20, 21]. 

Here, we detailed more specifically the clinical aspects of APS, enlightening its implications on fertility, pregnancy, and fetal development.

### 2.1. On the Mother's Side

Pathologies linked to APS during pregnancy include recurrent thrombotic events (RTEs) as well as specific obstetrical pathologies. The coexistence of both thrombosis and miscarriage is estimated at 2.5–5% of APS pregnancies [19].

RTEs are major problems during pregnancy because of the management they implicate and the risk of complications, such as pulmonary embolism (PE). However, thrombotic events are usually low under adequate medications in APS patients with ongoing pregnancy. Interestingly, the Nimes Obstetricians and Hematologists Antiphospholipid Syndrome (NOH-APS) observational study compared the incidence of thrombotic events in 517 women with purely obstetrical APS to 796 seronegative women with a history of pregnancy loss. The annual rate of thrombotic complications, defined by deep-venous thrombosis (DVT, 1.46%), PE (0.43%), superficial vein thrombosis (0.44%), and cerebrovascular events (transient ischemic attack and stroke, 0.32%), was found to be higher in obstetrical APS women than in control patients (resp., 0.43%, 0.12%, 0.14%, and 0.09%) [4].

Furthermore, in more than 20% of cases, APS in pregnancy may present with minor symptoms such as thrombocytopenia or livedo reticularis [19]. Low platelet counts (<100 G/L) can be difficult to deal with, especially under LMWH treatment. Therefore, special attention and close follow-up should be considered. Livedo reticularis is an affection of the skin with persistent, not reversible with rewarming, violaceous, red or blue, reticular or mottled, pattern of the skin of trunk, arms or legs [6]. This condition could be explained by a decrease in blood flow in dermic venules, partly due to microthrombosis and inflammation of vessel wall. 

More specific obstetrical manifestations include severe preeclampsia, which is defined in Table 2. Preeclampsia generally affects 2–8% of pregnancy [1]. A cross-sectional study conducted in Florida on 141 286 women who delivered in 2001 showed that women with high aPL titers (*n* = 88) had an increased risk of preeclampsia or eclampsia (adjusted odds ratio or AOR 2.93), placenta insufficiency (AOR 4.58), and prolonged length of stay at hospital (>three days, AOR 3.93) [22]. 

Complications of preeclampsia include various rarer conditions such as eclampsia and hemolysis, elevated liver enzymes, and low platelet count (HELLP) syndrome. Incidence of HELLP syndrome in APS patients is difficult to determine; however it seems more severe and occurs earlier in pregnancy than in patients not affected by APS [1, 5]. 

Finally, mothers can also be affected by catastrophic APS (CAPS). CAPS represents 1% of APS and can occur outside of pregnancy. CAPS is defined as a “thrombotic storm” secondary to microangiopathic diffuse thrombosis leading to multiorgan failure. 6% of CAPS seems to be associated with pregnancy and postpartum, but this is probably underestimated [23].

CAPS differential diagnosis can be difficult and large during pregnancy, including HELLP syndrome, thrombocytopenic thrombotic purpura (TTP), and disseminated intravascular coagulation (DIC). Since both mother and fetal outcomes are engaged, early diagnosis and management of CAPS are crucial. CAPS is indeed fatal in about 50% of cases even once aggressive therapy is started [1, 23].

### 2.2. On the Fetus' Side

aPL is responsible for fetal development and growth impairments and can affect any stages of pregnancy. 

In the general population, miscarriages affect about 1 to 4-5 pregnancies; however, recurrent pregnancy losses (RPLs) represent only 1% of pregnancy. Although fetal chromosomal abnormalities are the main cause of this condition, aPL is found in 15% of recurrent fetal losses, implicating that APS is one of the main acquired cause for recurrent miscarriages [8]. 

Stillbirth is a really rare condition in pregnancy in industrialized countries. However, in the “Euro-Phospholipid” project on 1000 patients, it affects up to 7% of APS pregnancies [19]. In the same study, IUGR due to placental insufficiency affected 11% of pregnancies and prematurity was found in 28% of pregnancies.

### 2.3. Other Manifestations

#### 2.3.1. Implantation Studies

Infertility and APS have been a controversial matter of study through the past years. The incidence of aPL in women with unexplained infertility and *in vitro* fertilization (IVF) failure seems significantly increased compared to control patients [24]. However, because of poorly designed studies, there is still a lack in evidence of aPL prediction on implantation or IVF outcome [25, 26]. Moreover, no study has clearly shown whether aPL could be associated with infertility so far, and precaution should be taken while interpreting positive aPL test results [24, 25]. 

#### 2.3.2. On the Infant's Side

In a prospective European multicenter registry, 134 babies born from mothers affected by APS have been followed up for 5 years (2005–2010); both clinical and biological parameters were analyzed [27].

 If no child presented thrombotic episodes, 3% of them (4/134) had neuropsychological development disorders, among which one autism was diagnosed. The conclusion of the study was that these development disorders were more common in these children and that specific and close follow-up should be given. 

These results should be interpreted with great caution. Because of the difficulty of diagnosis and the frequent changes in the current definition, the general population prevalence in autism is only estimated at around 1% of children [28], suggesting that the association between APS mothers and autistic children is hard to believe. 

**Figure 1:**
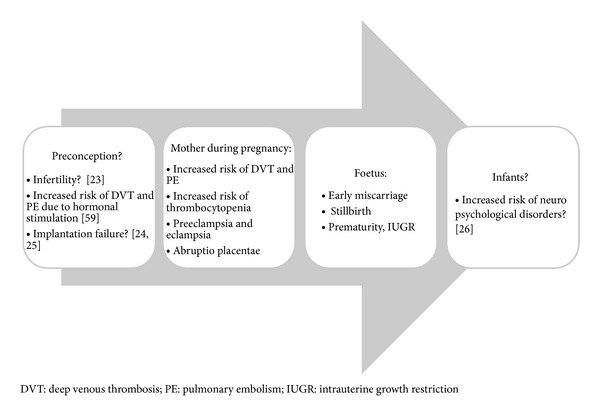
Obstetrical APS pathologies: more than a single disease.

Moreover, the presence of aPL in these children is estimated at 20%, with no association with any specific clinical manifestation of APS or SLE. Long-term consequences should be evaluated prior to give further conclusion [27].  Figure 1 summarizes the different clinical manifestations of obstetrical APS described above. 

## 3. Pathogenesis of aPL during Pregnancy

In APS, aPL binds to endothelial cells, platelets, and monocytes, inducing a proinflammatory and prothrombotic state responsible for thrombotic complications [29]. During pregnancy, aPL targets the placenta, especially the cytotrophoblastic cells (CT). Initially, the CT differentiates into two cell types. On one hand, the villous trophoblast will fuse to form the syncytiotrophoblast (ST), a barrier of protection between the mother and the fetus. On the other hand, the extravillous trophoblast (EVT) will progressively invade and colonize the maternal endometrium [30]. 

aPL's main antigen is *β*2GP1, a cationic protein that is normally in a “closed conformation” when free in the plasma of patients. It is composed of five homologous domains of approximately 60 amino acids each. Domains I and V are the two domains positively charged [31, 32]. During normal pregnancy and ST formation, anionic phospholipids are externalized at trophoblastic cell surface, leading to the binding of *β*2GP1 via domain V. This binding offers a potential site of actions for aPL by changing the conformation of the protein from a circular to an open form and exposing domains I to IV to the surface [9, 10, 31, 32]. In 2009, an international multicenter study tested 477 anti*β*2GP1 antibody positive plasma samples for antibodies specific for domain I of *β*2GP1. It showed a stronger association of these specific antibodies with obstetrical morbidity compared to total anti*β*2GP1 IgG antibodies (odd ratio 2.4; [1.4–2.5], 95% confidence interval). However, further studies need to be performed to add this test to obstetrical APS criteria [33].

Pathogenesis of aPL on trophoblastic cells is a matter of debate and several hypotheses have been succeeding through time. 

It has first been hypothesized that, as a parallel to the “thrombotic APS,” obstetrical APS was mainly linked to thrombosis. As proof, histological analysis of placenta collected from spontaneous abortions (*N* = 15), fetal deaths (*N* = 13), and live births (*N* = 16) from APS patients was found to have more thrombotic characteristics as compared to control placenta. However, these findings were not specific for APS, as placenta collected from women with clinical characteristics of APS but without aPL has the same histological findings [34]. 

Moreover, inflammation, including fibrin deposits, was more represented than thrombosis in histological analysis of placenta of APS women, suggesting another mechanism in pregnancies affected by APS [35]. 

Studies were then more focused on inflammation processes by aPL on trophoblastic cells and this role was confirmed by *in vitro and in vivo *studies [36–40]. Initiation of complement cascade by aPL and increase in C4 deposition in placenta of mice treated with aPL were strongly linked to adverse fetal outcome [36–39]. Moreover, both C4 and C5 deficient mice were protected from fetal injury when treated with aPL IgG [40].

More recently, immunomodulation has shown to play a critical role in APS. Implications of Toll-like receptors (TLRs) in autoimmune diseases offered a new perspective for the understanding of APS. TLR is a family of 10 different receptors identified in humans and is responsible for the innate immune response. They recognize specific sequences conserved in pathogens; and the main ones are considered to be TLRs 2 and 4 [41]. In thrombotic APS model, TLR 2 and TLR 4 have both been implicated in the pathological activation of endothelial cells, monocytes, and platelets [42–48]. More recently, aPL has been shown to induce both translocations of TLR 7 and TLR 8 in the endosomes of human monocytes, sensitizing both receptors to their specific ligands [49]. In obstetrical APS, TLR 4 has been implicated in the pathological activation of HTR-8 cell line, an EVT cell line, by aPL, leading to an uncontrolled inflammation and apoptosis [50]. 

Immunomodulation by TLR offered a new insight on how aPL triggered placental alteration. Thus, it has been shown that aPL could mediate a nonthrombotic noninflammatory trophoblast modulation, by altering directly their own properties. Trophoblastic properties implicate three different mechanisms, defined as (a) migration, (b) invasion, and (c) differentiation [51–53].

First, Mulla et al. showed migration alteration of first trimester trophoblastic cells by monoclonal anti*β*2GP1 antibodies by decreasing IL-6 secretion and signal transducer activator of transcription 3 (STAT3) protein expression [54]. 

Invasion and proliferation impairments by aPL have also been studied *in vitro*. aPL has been shown to prevent HTR-8, a trophoblastic cell line, from invading on matrigel assay and to decrease integrins proteic expressions [55].

Finally, it has previously been described that antiphosphatidylserine antibodies, a type of aPL that is not part of the definition of APS, were responsible for syncytiotrophoblast fusion impairments [56]. A decrease in *β*-human choriogonadotropin (hCG) secretion, a hormone normally produced by ST, has also been described in term placenta incubated with high doses of anti *β*2GP1 antibodies [57]. In BeWo cell, a choriocarcinoma cell line, we recently showed that anti*β*2GP1 Abs significantly decrease cell differentiation in a dose-dependent way and that this effect was reversed by decreasing TLR 4 membranous expression (manuscript under submission).

Trophoblastic cells seem not the only cell type affected by aPL. Impaired endometrial differentiation in decidual phenotype as well as endometrial angiogenesis inhibition by aPL has also been advocated [58]. Laboratory findings on endometrial cells were different from those found on other cell types. Anti*β*2GP1 antibodies purified from APS patients were found to inhibit angiogenesis, VEGF secretion, and NF*κ*B activation in a dose-dependent way in endometrial cells [13, 14]. This implicates that pathological mechanisms of aPL can differ between various cell types which could explain variations in treatment efficiency. 

*Pathogenesis of Antiphospholipid Antibodies in Pregnancy*.

(1) Mechanisms on placental cell

- Thrombosis
  - Aspecific mechanism [30]
- Inflammation
  - Complement activation [31, 33–36]
- Immunomodulations
  - TLR 4 activation by aPL [42]
- Defective placentation
  - Migration: decrease in IL-6 and STAT3 expression [50]  
  - Invasion: decrease in integrin expression [51]
  - Differentiation: decrease in *β*-hCG secretion [52] and decrease in fusion [53]

(2) Mechanisms on endometrial cells [13, 14]

- Angiogenesis inhibition
- Decrease in VEGF secretion
- NF*κ*B activation inhibition.

## 4. Treatments and Future Perspectives

**Figure 2:**
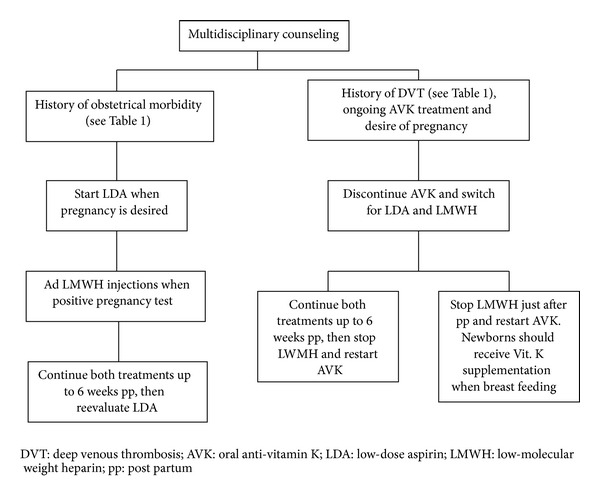
Obstetrical APS first-line management.

APS pregnancies are real challenges for clinicians and therefore should be planned. Careful counseling is required and multidisciplinary management is the key to a successful pregnancy [2, 59]. APS patients already under oral anticoagulant drugs should be informed of potential teratogenic effects. Once pregnancy is confirmed, oral anticoagulation should be immediately stopped and switched to low-molecular weight heparin (LMWH) for the rest of the pregnancy. Guidelines for first-line APS treatments during pregnancy vary between countries. However, combination of low-dose aspirin (LDA) and LMWH injections is usually admitted and improves both fetal and mother outcomes [60] (Figure 2). Thus, without treatment, the chances of successful pregnancy are around 30%, 50% with LDA alone, and up to 70% with both molecules [61]. 

Treating infertile patients with positive aPL is a matter of debate. Studies conducted on LDA and LMWH indications in “aPL infertility” showed contradictory results. Even if heparin seems to improve implantation, there is still no evidence that these two treatments are truly effective for this indication [62, 63]. 

Biological roles of both aspirin and heparin are large. Nishino et al. have shown that aspirin could decrease thromboxane A2 production and prostaglandin I2 formation, two molecules implicated in pregnancy hypertension and preeclampsia [64]. More recently, aspirin has also been shown to upregulate interleukin-3 (IL-3) production. This molecule seems necessary for trophoblast invasion and placental formation [65].

Heparin actions have been summarized by Kwak-Kim et al. [66]. Heparin as LMWH are anticoagulant molecules that prevent clot formation and can be safely used during pregnancy. However, their roles are not limited to their antithrombotic properties. Among them, they have also been shown to be antiinflammatory and anti-apoptotic molecules. 

Both molecules have also their limitations. Mulla et al. showed that neither heparin nor LMWH could reverse the effects of anti*β*2GP1 Abs on trophoblast migration [54]. 

This could partly explain treatment failure in 30% of APS pregnancies. For them, the literature is poor with no evidence-based management defined. Second-line treatments include steroids, hydroxychloroquine (HCQ), intravenous immunoglobulin injections, and plasmaphereses [67, 68]. Among them all, HCQ is the safest molecule used in pregnancy [69]. This antimalarial drug is commonly used in lupus patients and has been shown to improve fetal outcome and to reduce lupus flares [16, 17]. Biologically, HCQ reduces the binding of anti*β*2GP1 Abs at the surface of trophoblastic cells [70]. Moreover, the expression of annexin A5, an anticoagulant molecule normally present at the trophoblastic cell surface, is reduced by anti*β*2GP1 Abs. HCQ has been shown to restore its expression, preventing the pathological activation of the trophoblastic cells [71]. We also have demonstrated that HCQ restored the effects of anti*β*2GP1 Abs on BeWo cell differentiation and decreased TLR 4 expression (manuscript under submission).

New molecules are also in development. As TLRs have been implicated in the pathological activation of different cell types in APS, specific p38 mitogen-protein kinase (p38-MAPK) and nuclear factor-*κ*B inhibitors, two molecules implicated in intracellular signaling by aPL via TLR, have been developed [72]. However, their uses in pregnancy seem limited by the fact that the suppression of innate immunity could lead to immunosuppression and poor fetal and mother outcomes.

Finally, special attention should be given to prevention of CAPS. As this rare condition can be fatal in about 1/2 of the cases despite any treatment, prophylaxis is still the most important way to avoid IT. Asherson defined special circumstances during which APS patients required special attentions [73]:

1. infections in APS patients should always be treated carefully;
2. when surgery is needed, APS patients should receive parenteral anticoagulation;
3. during postpartum, women should continue anticoagulation for 6 weeks.

## 5. Take-Home Messages

- Obstetrical APS is an entity with high pregnancy complications for both mother and fetus.
- Counseling, multidisciplinary management, and tight follow-up are the keys to successful pregnancy.
- Screening for high-risk APS patients is necessary to improve their pregnancy outcomes.
- A better understanding of pathological mechanisms is necessary for therapeutic improvement.

## Figures and Tables

**Table 1:** Criteria of obstetrical APS [3]. APS is diagnosed when at least one of the following clinical criteria and one of the following laboratory criteria are met.

Clinical criteria	Biological criteria
(i) 3 or more consecutive spontaneous abortions before the 10th WG*, with maternal anatomic or hormonal abnormalities and paternal and maternal chromosomal causes excluded	(i) Lupus anticoagulant (LA) present in plasma, on two or more occasions at least 12 weeks apart, detected according to the guidelines of the International Society on Thrombosis and Haemostasis
(ii) One or more unexplained deaths of a morphologically normal fetus at or beyond the 10th WG*, with normal fetal morphology documented by ultrasound or by direct examination of the fetus	(ii) Anticardiolipin (aCL) antibody of IgG and/or IgM isotype in serum or plasma, present in medium or high titer, on two or more occasions, at least 12 weeks apart, measured by standardized ELISA
(iii) One or more premature births of a morphologically normal neonate before the 34th week of gestation because of eclampsia or severe preeclampsia or recognized features of placental insufficiency**	(iii) Anti*β*2glycoprotein-1 antibody of IgG and/or IgM isotype in serum or plasma (in titer >99th percentile), present on two or more occasions, at least 12 weeks apart, measured by standardized ELISA

*WG: week of gestation.

**Placental insufficiency features include abnormal or nonreassuring fetal surveillance test, abnormal Doppler flow velocimetry waveform analysis suggestive of fetal hypoxemia, oligohydramnios, and postnatal birth weight less than the 10th percentile for the gestational age.

**Table 2:** Preeclampsia criteria.

Preeclampsia	(i) High blood pressure (>140/90 mmHg) associated with proteinuria (300 mg in a 24-hour urine sample) after 20 WG or (ii) increase in SBP ≥30 mmHg or in DBP ≥15 mmHg after 20 WG, with edema and/or proteinuria

Severe preeclampsia	(i) Presence of preeclampsia as described above and at least one of the following criteria (ii) SBP ≥160 mmHg, or DBP ≥110 mmHg on two occasions at least 6 hours apart (iii) Proteinuria ≥5 g in a 24-hour urine sample collected at least 4 hours apart (iv) Pulmonary edema or cyanosis (v) Oliguria (<400 mL in 24 hours) (vi) Persistent headaches (vii) Epigastric pain and/or impaired liver function (viii) Thrombocytopenia (ix) Oligohydramnios, decreased fetal growth, or placental abruption

## Abbreviations

TLR 4: Toll-like receptor 4
aPL: Antiphospholipid antibodies
STAT3: Signal transducer activator of transcription 3
hCG: Hormone chorionic gonadotrophin
VEGF: Vascular endothelial growth factor
NF*κ*B: Nuclear factor-kappa B.
